# First molecular isolation of *Mycoplasma* ovis from small ruminants in North Africa

**DOI:** 10.4102/ojvr.v82i1.912

**Published:** 2015-06-08

**Authors:** Mohamed R. Rjeibi, Mohamed A. Darghouth, Houda Omri, Khemaïs Souidi, Mourad Rekik, Mohamed Gharbi

**Affiliations:** 1 Laboratoire de Parasitologie, Université de la Manouba, École Nationale de Médecine Vétérinaire de Sidi Thabet, Tunisia; 2 International Centre for Agricultural Research in the Dry Areas, Amman, Jordan

## Abstract

Eperythrozoonosis is a small ruminant disease caused by the bacterium *Mycoplasma ovis* (formerly known as *Eperythrozoon ovis*). Whilst acute infection in sheep may result in an anaemia and ill thrift syndrome, most animals do not develop clinical signs. Molecular methods were used to compare and evaluate the prevalence of infection with *M. ovis* in sheep and goats in Tunisia. A total of 739 whole blood samples from 573 sheep and 166 goats were tested for the *M. ovis* 16S rRNA gene using PCR. The overall prevalence was 6.28% ± 0.019 (36/573). Only sheep were infected with *M. ovis* (*p* < 0.001), and the prevalence was significantly higher in central Tunisia (29.2%) compared with other regions (*p* < 0.05). The prevalence revealed significant differences according to breed and bioclimatic zones (*p* < 0.001). Furthermore, the prevalence in young sheep (35/330; 10.6%) was higher than in adults (1/243; 0.41%) (*p* < 0.001). Only sheep of the Barbarine breed were infected, with a prevalence of 11.8% (*p* < 0.001). This is the first molecular study and genetic characterisation of *M. ovis* in North African sheep breeds.

## Introduction

There are over 1.07 billion sheep in the world, 27% of which are located in Africa (Food and Agriculture Organization of the United Nations 2010). Sheep are amongst the major economically-important livestock species in Tunisia, with a total population of 6.955 million heads; they play an important role in the livelihood of resource-poor farmers (Observatoire National de l'Agriculture 2006). In Tunisia, as in several other North and sub-Saharan African countries, small ruminants face harsh production conditions, which are exacerbated by both a high frequency of extreme climatic conditions and a plethora of bacterial and viral diseases with high prevalence rates of infections, such as brucellosis, border disease and peste des petits ruminants. Several parasitic diseases occur with high incidence and infection intensity, such as echinococcosis (Lahmar *et al.* 2013), fasciolosis (Akkari, Gharbi & Darghouth 2011), gastrointestinal helminths (Akkari, Gharbi & Darghouth 2012), toxoplasmosis (Gharbi *et al.* 2013) and piroplasmosis (M'ghirbi *et al.* 2013; Rjeibi, Darghouth *et al.* 2014; Rjeibi, Gharbi *et al.* 2014). This results in a very high challenge to the immune system, which is further exacerbated by long periods of food shortage during which quantitative and qualitative nutrient supplies are well below animals’ requirements (Rekik, Aloulou & Ben Hamouda 2005). All of these factors increase the impact of several pathogens.

The local sheep and goat populations in North Africa are subject to high exposure to new pathogens such as *Theileria lestoquardi* (Rjeibi, Darghouth *et al.* 2014) because of the high permeability of its borders to important flows of small ruminants from both the western and southern neighbouring countries.

*Mycoplasma ovis* (*Eperythrozoon ovis*), is a haemotropic mycoplasma that attaches to the red blood cells of sheep; it was first described by Neitz in 1934 (Neitz, Alexander & Du Toit 1934). *Mycoplasma ovis* infection of sheep may cause mild to severe anaemia with a high lethality rate in lambs, whereas adults are usually chronically infected but show no clinical signs, with a low lethality rate (Grazziotin *et al.* 2011). *Mycoplasma ovis* infection is transmitted by blood-feeding arthropods and has been reported in domestic small ruminants: sheep and goats (Hornok *et al.* 2012; Neimark, Hoff & Ganter 2004), in white-tailed deer (*Odocoileus virginianus*) and reindeer (*Rangifer tarandus*) (Boes *et al.* 2012; Stoffregen *et al.* 2006). *Mycoplasma ovis* was detected for the first time by PCR in Germany in sheep by Neimark *et al.* (2004); it has also been reported in Hungary (Hornok *et al.* 2009), Japan (Tagawa *et al.* 2012) and the USA (Deshuillers *et al.* 2014).

The 16S rRNA gene of this organism was sequenced, as described by Grazziotin *et al.* (2011), to determine the genetic relationship between this and other haemotropic bacterial species. Phylogenetic analysis revealed that this wall-less bacterium is not a rickettsia but rather a mycoplasma and is closely related to several other uncultivated, epi-erythrocytic mycoplasmas comprising a recently-identified group, namely the haemotropic mycoplasmas. This group consists of former *Eperythrozoon* and *Haemobartonella* species as well as the newly-identified epi-erythrocytic mycoplasmas (Neimark *et al.* 2004). The authors present the first molecular study and genetic characterisation of *M. ovis* in Africa from small ruminants in different regions of Tunisia (North Africa).

## Research method and design

### Small ruminant population

**FIGURE 1:**
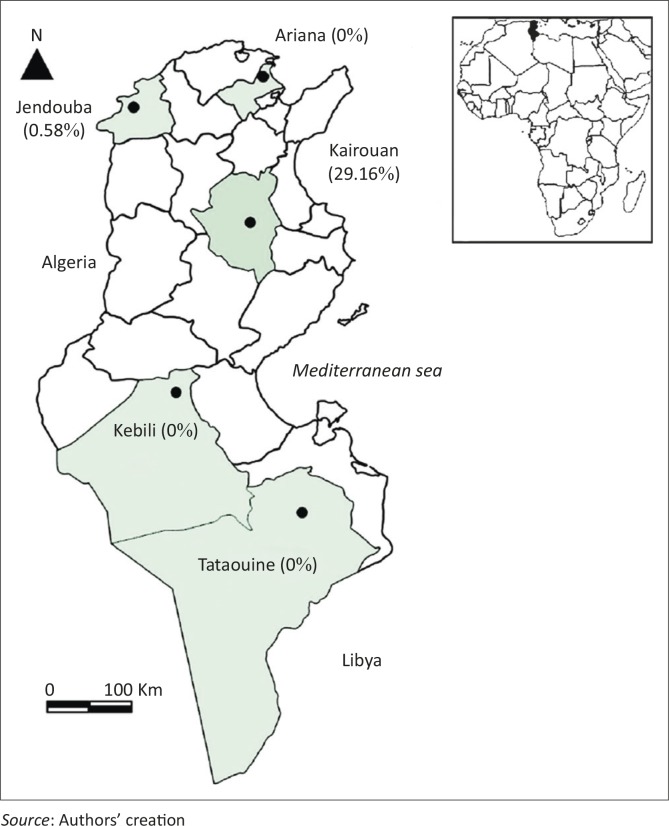
Sheep *Mycoplasma ovis* molecular prevalence in five Tunisian localities. Prevalence, as percentage, indicated between parentheses.

This cross-sectional study was carried out on five farms located in four different Tunisian bioclimatic zones (humid, semi-arid, arid and Saharan) (Figure 1). Data related to annual rainfall, temperature, moisture and altitude in the different sampling sites are reported in Table 1. All the farms were private and traditionally managed. In Ariana and Jendouba, the production system is mainly livestock-forage integrated. In Kairouan, the production system is crop-livestock relying on the use of barley and further south, in Kebili and Tataouine, the system is extensive rangeland-based. Animals mainly grazed and received various levels of supplementation (barley and bran), especially during summer and autumn. A total of 739 small ruminants consisting of 573 sheep (303 Barbarine and 270 Queue Fine de l'Ouest) and 166 goats (144 of the local breed and 22 crossbred animals) were randomly included in the survey (Table 1). The genetic type was determined according to the description of Rekik *et al.* (2005). Because of the lack of information on animals, they were ranked in two age groups: less and more than one year. EDTA whole blood samples were collected from each animal and stored at −20 °C until used. All sheep included in the current survey were examined for ticks, which were collected into labelled tubes containing 70% ethanol. The ticks were identified under a stereomicroscope based on the key described by Walker *et al.* (2003). Three tick infestation indicators were determined:

- Infestation prevalence (%) = 100 × number of infested animals/total number of examined animals.
- Infestation intensity = number of ticks/number of infested animals.
- Abundance = number of ticks/total number of examined animals.

**TABLE 1:** Geographic and abiotic characteristics of the studied Tunisian areas and corresponding *Mycoplasma ovis* prevalence in sheep.

Region	Governorate	Farm sheep population	Sampled sheep	Bioclimatic zone	Mean altitude§	MAT† (°C) (Min–Max)§	MAP‡ (mm)§	Relative humidity (**%**) (Min–Max)§	Prevalence (**%**)
North	Ariana	220	80	Semi-arid	25	18.4 (7–33)	450	39–92	0
Jendouba	534	172	Humid	800	18.1 (8–31)	1029	31–100	0.58
Centre	Kairouan	400	120	Arid	68	19.5 (6–37)	308	28–95	29.16*
South	Kebili	350	166	Saharan	43	21.4 (6–46)	100	14–82	0
Tataouine	150	35	Saharan	247	20.5 (6–38)	51	15–98	0

†, Mean Annual Temperature; ‡, Mean Annual Precipitation; §, Climatic data were gathered from Weather Online (n.d.) and Climatedata.eu (n.d.).

*, *p* < 0.05

### Polymerase chain reaction

DNA was extracted from 300 µL of whole blood using the Wizard® Genomic DNA purification kit (Promega, Madison, USA) according to the manufacturer's instructions and stored at −20 °C until used. The PCR assay for *M. ovis* DNA detection was performed using the protocol described by Grazziotin *et al.* (2011), with a set of primers that amplifies a 1341 bp region of the *M. ovis* 16S rRNA gene. The forward primer was 16S Fw (5’-ATG CAA GTC GAA CGA GTA GA-3’) and the reverse primer was 16S Rv (5’-TGA TAC TTT CTT TCA TAG TTT G-3’). The PCR mixture consisted of 5 µL of 5X PCR buffer (50 mM Tris–HCl, pH 8.5; 50 mM NaCl), 1.5 mM MgCl_2_, 0.2 mM of each dNTP (dATP, dCTP, dGTP, dTTP), 10 pmol of each primer, 1.25 U *Taq* Polymerase (Vivantis, USA), distilled water and 5 µL of DNA template. The DNA amplification was performed using the following programme: 4 minutes’ denaturation at 95°C, followed by 39 cycles (94°C for 1 min, 51.6°C for 30 s and 72°C for 90 s) and a final extension at 72°C for 5 min (Grazziotin *et al.* 2011).

### DNA sequencing and phylogenetic analyses

The Wizard SV gel and PCR clean-up system (Promega, USA) was used to purify five selected PCR products according to manufacturer's instructions. The fragments were sequenced in both directions, using the same primers as for PCR. A conventional Big Dye Terminator cycle sequencing ready reaction kit (Applied Biosystems, Foster City, CA) and an ABI3730XL automated DNA sequencer were used. The chromatograms were evaluated with ChromasPro software (version 1.7.4; Technelysium Pty Ltd 2012). The MEGA 5.1 software programme was used, as described by Tamura *et al.* (2011), to perform the sequence alignments, after which the GenBank database was used to compare the sequences by means of nucleotide sequence homology. Searches were made at the network server of the National Center for Biotechnology Information (NCBI) using BLAST. The phylogenetic tree was constructed by the neighbour-joining method. The dataset was resampled 1100 times to generate bootstrap values.

### Statistical analysis

Prevalence was compared using the chi-square test with Epi Info 6 software (Dean *et al.* 2011). For major sources of variation (species, breeds, bioclimatic zones, age group), a probability below 0.05 was used as a threshold for statistical significance.

## Results

### Tick infestation

A total of 282 adult ticks were collected from 573 sheep (143 males and 139 females). The sex ratio (M:F) was 1.03. These ticks belonged to two genera – *Hyalomma* and *Rhipicephalus* – and included *Hyalomma excavatum, H. dromedarii, Rhipicephalus turanicus, R. sanguineus* and *R. camicasi*. *Rhipicephalus turanicus* was the dominant species (45.03%), followed by *H. excavatum* (41.84%) (*p* < 0.001). The overall prevalence of infestation had a value of 20.4% (117/573). The highest prevalence was observed for *R. turanicus* (12.04%), followed by *Hyalomma excavatum* (5.41%) and the lowest was reported for *R. camicasi* (0.35%) (*p* < 0.001). The overall intensity and abundance of infestation were 2.41 and 0.49 ticks/animal respectively. The highest intensity of infestation was observed for *H. excavatum* (3.81 ticks/animal), followed by *H. dromedarii* (2.75) and the lowest was reported for *R. camicasi* (1 tick/animal). The most abundant tick species was *R. turanicus* (0.22), followed by *H. excavatum* (0.2), whilst *R. camicasi* was the least abundant tick (0.003) (Table 2). The association between tick burden and prevalence of *M. ovis* was not significant (*p* > 0.05).

**TABLE 2:** Tick infestation indicators.

Tick genera	Tick species	Number of ticks	Prevalence of infestation		Intensity of infestation	Abundance
			n	%		
Rhipicephalus	Rhipicephalus turanicus	127	69/573	12.04	1.84	0.22
	Rhipicephalus sanguineus	13	7/573	1.22	1.86	0.02
	Rhipicephalus camicasi	2	2/573	0.35	1	0.003
Hyalomma	Hyalomma excavatum	118	31/573	5.41	3.81	0.2
	Hyalomma dromedarii	22	8/573	1.4	2.75	0.04
**Overall**	**-**	**282**	**117/573**	**20.4**	**2.41**	**0.49**

### Molecular prevalence of *Mycoplasma ovis* in Tunisian sheep and goats

*Mycoplasma ovis* was only detected in 36 out of 573 sheep (6.28% ± 0.019). The subsequent epidemiological indicators were estimated in sheep since none of the goats were infected with *M. ovis* (*p* = 0.0009). The regional prevalence in sheep differed significantly; the highest rate was observed in Kairouan (35/120; 29.2% ± 0.081), followed by Jendouba (1/172; 0.58% ± 0.0017) and zero in Ariana (0/80), Kebili (0/166) and Tataouine (0/35) (*p* < 0.001). There were no statistically significant differences in the prevalence of *M. ovis* in tick-infested and non-infested sheep (*p* > 0.05). The *M. ovis* infection prevalence was significantly higher in young sheep (35/330; 10.6%) than adults (1/243; 0.41%) (*p* < 0.001). Only Barbarine sheep were infected, with a prevalence of 11.8% (*p* < 0.001).

### Sequence of *Mycoplasma ovis* 16S rRNA gene

*Mycoplasma ovis* infection was confirmed by sequencing 16S rRNA from five randomly selected positive samples: one from Jendouba (North Tunisia) and four from Kairouan (Central Tunisia). Comparison of the partial sequences of 16S rRNA gene (884 bp length) revealed 99.6% homology between the sequence from Jendouba (KJ458989) and the sequence from Kairouan (KJ458990). These two sequences showed a difference of three nucleotides at positions 238 (G/A), 731 (T/C) and 822 (T/C). The four isolates sequenced from Kairouan had 100% homology.

**FIGURE 2:**
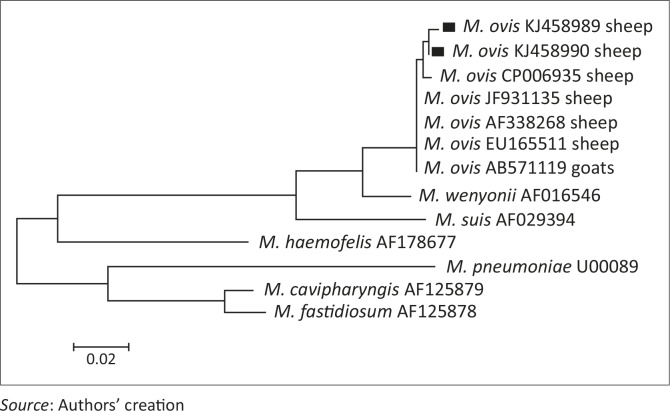
Partial sequence 16S rRNA gene phylogenetic tree of *Mycoplasma ovis* identified in this survey and the main *Mycoplasma* species. The tree was constructed using the neighbour-joining method. The percentage of replicate trees in which the associated taxa clustered together in the bootstrap test (1100 replicates) was shown next to the branches (Felsenstein 1985). The evolutionary distances were computed using the Tamura-Nei method (Tamura & Nei 1993) and are in the units of the number of base substitutions per site. Evolutionary analyses were conducted in MEGA5.1 (Tamura *et al.* 2011). Species described in this study are indicated with a black square.

Novel *M. ovis* 16S rRNA genotypes named MOTNSH01 and MOTNSH02 (GenBank accession number KJ458989 and KJ458990 respectively) were identified in this study. These sequences derived from northern and central Tunisia respectively; they showed 99% homology to *M. ovis* in Germany (AF338268) (Neimark *et al.* 2004), Hungary (EU165511) (Hornok *et al.* 2009), Japan (JF931135) (Tagawa *et al.* 2012) and the USA (CP006935) (Deshuillers *et al.* 2014) (Figure 2).

## Ethical considerations

This study was conducted in accordance with relevant national and international guidelines on handling animals, taking care to respect animal welfare.

## Trustworthiness

The experiment was considered to be both reliable and valid. Reliability of the study was tested by PCR using positive control DNA obtained from sheep representing the disease and confirmed by sequencing. The experimental procedures were performed with care and interpretation of results was done according to the established standards.

## Discussion

*Mycoplasma ovis* is a causative agent of haemolytic anaemia in small ruminants aged 4 weeks and older (Aguirre *et al.* 2009; Neimark *et al.* 2004), resulting in small but widespread persistent economic losses. The effect of *M. ovis* infection is more severe in stressful conditions when animals are infected by internal parasites or other pathogens and/or are undernourished; death may occur in severely infected lambs (Ohtake *et al.* 2011). *Mycoplasma ovis* usually causes latent infections; carriers are more susceptible to secondary infections such as those caused by *Streptococcus* spp., *Pasteurella* spp. and *Eimeria* spp. (Song *et al.* 2014).

The present molecular study was carried out to investigate *M. ovis* infection in small ruminants in Tunisia. The overall prevalence (6.28%) was lower than sheep in Hungary (51.5%) (Hornok *et al.* 2009), Argentina (81.8%) (Aguirre *et al.* 2009) and goats in China (41%) (Song *et al.* 2014). All goats sampled were negative; this could be because of: (1) a higher resistance of goats to *M. ovis*, (2) insufficient sample size to detect low infection prevalence or (3) the low sensitivity of the technique to very low *Mycoplasma* burdens. Further studies are needed to explore these hypotheses. The bacteria were detected in central Tunisia (arid region), with relatively high molecular prevalence (29.2%). This regional discrepancy is difficult to explain but it could result from the high small ruminant density in the studied region compared to other regions, resulting in high ectoparasite burdens. Indeed, during all the visits to the farms, the presence of large numbers of biting *Diptera* populations was observed (results not shown). Genetic analyses showed that the analysed amplicons are closely related to all GenBank *M. ovis* sequences isolated from small ruminants, forming an independent clade. The two sequences (884 bp length) presented a difference of three base pairs; it is difficult to attribute this difference to the distance between the two regions (243 km) or to heterogeneity in these populations.

Few data are available about *M. ovis* infection in the international literature; the clinical significance of this *Mycoplasma* spp. infection should be studied further. Despite the presence of an intensive uncontrolled animal trade in Tunisia, a very large difference in the prevalence was observed. The lack of information in the North African region and other surrounding sub-Saharan countries is an important gap that should be filled.

## Limitations of the study

The study did not cover the whole of Tunisia; it was limited to five governorates and the findings are thus not generalisable to the country as a whole.

### Recommendations

It is recommended that further studies be conducted in order to get an idea about the impact of this pathogen on small ruminants’ health.

## Conclusion

The present findings should encourage animal health decision makers to: (1) implement regional epidemiological surveys concerning *M. ovis* infection and (2) implement a regional animal health monitoring system to detect any increase in the prevalence of arthropod-borne diseases. Field veterinarians should include this pathogen in the differential diagnosis of arthropod-borne pathogens. The zero prevalence in the local goat population should be investigated further.
